# miR-182 promoter hypermethylation predicts the better outcome of AML patients treated with AZA + VEN in a real-world setting

**DOI:** 10.1186/s13148-025-01823-1

**Published:** 2025-02-05

**Authors:** Yilan Xu, Danyang Li, Na Wang, Bei Ge, Chen Meng, Min Zhao, Zihan Lin, Min Li, Yigang Yuan, Yue Cai, Liuzhi Shi, Shenmeng Gao, Haige Ye

**Affiliations:** 1 Department of Hematology, The First Affiliated Hospital of Wenzhou Medical University, 1 Xuefubei Street, Ouhai District, Wenzhou, 325000 Zhejiang Province China; 2 Medical Research Center, The First Affiliated Hospital of Wenzhou Medical University, 1 Xuefubei Street, Ouhai District, Wenzhou, 325000 Zhejiang Province China; 3 Department of Blood Transfusion, The First Affiliated Hospital of Ningbo University, Ningbo, 315099 Zhejiang Province China; 4 Health Care Center, The First Affiliated Hospital of Wenzhou Medical University, 1 Xuefubei Street, Ouhai District, Wenzhou, Zhejiang Province China; 5 Infection Control Department, Eye Hospital, Wenzhou Medical University, 270 Xueyuanxi Road, Wenzhou, Zhejiang Province China; 6 Department of Clinical Medicine, Wenzhou Medical University, Ouhai District, Wenzhou, Zhejiang Province China

**Keywords:** 5-Azacytidine, Venetoclax, DNA methylation, Acute myeloid leukemia

## Abstract

**Background:**

5-Azacytidine (AZA) combined with the BCL2 inhibitor Venetoclax (VEN) is the standard treatment for elderly acute myeloid leukemia (AML) patients or those who are unfit for intensive chemotherapy (elderly or unfit AML). However, an effective and rapid predictive biomarker to predict treatment outcome remains elusive.

**Methods:**

miR-182 promoter methylation was measured in 94 AZA + VEN-treated elderly or unfit AML patients and 20 normal controls (NCs) samples. To determine whether miR-182 promoter methylation is a predictive marker of clinical outcomes in AZA + VEN-treated AML patients in a real-world setting, we analyzed and compared the complete remission (CR)/CR with incomplete hematologic recovery (CRi) rate, overall survival (OS), and leukemia free-survival (LFS) across different methylation groups: miR-182 promoter hypomethylation (median value < 20.21%) and hypermethylation (> 20.21%) in a retrospective study.

**Results:**

The average methylation frequency was markedly higher in 94 AZA + VEN-treated elderly or unfit AML patients than that in 20 NCs. However, some AML patients (11.7%) still presented low miR-182 promoter methylation (< 10%). The average time to obtain CR/CRi was shorter in AML patients with miR-182 promoter hypermethylation than AML with hypomethylation. Moreover, the median OS and LFS were longer in AML patients with miR-182 promoter hypermethylation than AML with hypomethylation. Finally, the area under the curve (AUC) for 1-year mortality was 0.831, for 2-year was 0.788, and for 3-year was 0.800.

**Conclusions:**

AML patients with miR-182 promoter hypermethylation have better outcomes. miR-182 promoter methylation is a predictive biomarker for AZA + VEN-treated AML patients.

**Supplementary Information:**

The online version contains supplementary material available at 10.1186/s13148-025-01823-1.

## Background

AML is predominantly considered a disease of the elderly with a median age of 68 [1]. Only approximately 30–40% of AML patients (age < 60) with non-M3 subtype survive for more than five years, while less than 20% of elderly AML patients survive for more than two years after chemotherapy treatment and bone marrow (BM) transplantation [2]. BCL2, an anti-apoptotic member of the BCL2 family, serves a main anti-apoptosis protein essential for the survival of leukemia stem cells (LSCs) [3]. Thus, targeting BCL2 by VEN results in the preferential elimination of LSCs [4]. Although single agent treatment with AZA or decitabine (DAC) treatment offers limited therapeutic benefits for elderly AML patients [5], the combination of AZA + VEN substantially improves the overall survival (OS) for elderly AML patients and has been approved by FDA as a standard therapy [6, 7].

Despite initially achieving a high response rate, AML patients treated with AZA + VEN commonly develop resistance over time [8]. The efficacy of VEN in AML patients is dependent on BCL2 expression levels. Thus, low expression of BCL2 protein in AML patients is strongly associated with VEN resistance [9]. For example, more differentiated monocytic AML cells commonly with low protein expression of BCL2 are refractory to VEN-based therapy [9]. In addition, TP53 inactivation [10], metabolic changes [11], and MCL1 activation [12] facilitate the resistance to VEN. In particular, AML cells, which depend on MCL1 but not BCL2 for survival, are resistant to VEN-based therapy [13, 14]. Therefore, there is an urgent need for the discovery of a biomarker to determine the sensitivity of AZA + VEN.

Our previous studies have demonstrated that miR-182 directly targets 3′-untranslated region of *BCL2* mRNA and decreases BCL2 protein expression by translational suppression in leukemic cells [15, 16]. However, miR-182 expression is silenced in AML cells because of miR-182 promoter hypermethylation. Thus, AML cells with miR-182 promoter hypermethylation have lower expression of miR-182 and higher expression of BCL2 protein than those with miR-182 promoter hypomethylation [16]. To further determine whether miR-182 promoter methylation is a predictive biomarker for AZA + VEN response in a clinical setting, we investigated the methylation frequency of miR-182 promoter in 94 AZA + VEN-treated elderly or unfit AML patients.

## Material and methods

### AML cell line

Human AML cell line U937 (ATCC, Manassas, VA, USA) was cultured in a humidified incubator with 5% CO_2_ at 37 °C in RPMI 1640 medium supplemented with 10% fetal bovine serum (Sigma‒Aldrich, St. Louis, MO, USA).

### Enrolled AML patients

We conducted a retrospective cohort study to review the untreated patients who were diagnosed with elderly AML (≥ 60 years) or young AML patients (< 60 years) who were unfit or unwilling to receive induction chemotherapy between January 2019 and December 2023 in the First Affiliated Hospital of Wenzhou Medical University. Acute promyelocytic leukemia (APL) was excluded in this study. 94 AML patients treated with AZA + VEN for at least one cycle and 20 NC samples were enrolled in this study. Our research was approved by the Institutional Ethics Committee of the First Affiliated Hospital of Wenzhou Medical University (KY2024-R011) and the *Helsinki Declaration* of 1975, as revised in 2013. All patients and healthy donors have provided informed consent for their participation. AML patients were diagnosed and classified according to the French–American–British (FAB) and the 2016 World Health Organization (WHO) criteria [17, 18].

### Leukemic cells from AML patients

Human BM mononuclear cells were isolated from untreated AML patients by Ficoll-Paque gradient (GE Healthcare Bio-Sciences, Pittsburgh, PA, USA) and deposited in liquid nitrogen until use. Primary AML samples were cultured at 37 °C in RPMI 1640 medium (Invitrogen, Carlsbad, CA, USA) with 10% fetal bovine serum (Invitrogen) in a humidified incubator with 5% CO_2_. AML samples were cultured in StemSpan SFEM medium supplemented with human recombinant interleukin-3 (IL-3), interleukin-6 (IL-6), and stem cell factor (SCF) at 10 ng/mL each. All procedures involving human participants were according to the ethical standards of the Ethics Committee of the First Affiliated Hospital of Wenzhou Medical University and the Declaration of Helsinki.

### Cytogenetic and molecular genetic analysis

Clinic hematological profiles and experimental examinations in AML patients were performed by routine methods [17]. Morphologic analysis of BM aspirate, flow cytometric immunophenotyping, cytogenetic analysis, and molecular examinations were performed to diagnose AML patients. At diagnosis, cytogenetic characteristics were analyzed using R-banded standard karyotyping and fluorescence in situ hybridization. Commonly, gene mutations tested by high-resolution melting analysis and direct DNA sequencing were carried out on BM mononuclear cells [19].

### Other procedures

For western blot, MethylTargetTM assay, bisulfite sequencing, treatment procedure, and definition of unfit AML patients, Engraftment of NOD/SCID‑IL2Rγ mice (NSG), primary AML blasts, drugs, and chemical reagents, viability assay, response criteria and outcomes, CD11b and CD14 staining by flow cytometry, and Wright‒Giemsa staining, please see the supplemental methods.

### Statistical analysis

Statistical analysis was performed with SPSS version 22.0 (SPSS, Chicago, IL, USA) and R software (v 4.2) (https://www.r-project.org/). Patients’ baseline characteristics were compared by independent t-test for numerical covariates with normal distribution or by Wilcoxon rank-sum test for numerical covariates without normal distribution. The chi-square test or Fisher’s exact test was performed for categorical covariates. The CR/CRi rate was compared across different miR-182 promoter methylation groups by Fisher’s exact test. The results of Kaplan–Meier estimates for OS and LFS across different miR-182 promoter methylation groups were compared using the log-rank test. Covariates related with OS or LFS with *P* < 0.05 in univariate analyses were enrolled in a subsequent multivariate Cox proportional analyses in which the impact of each covariate was adjusted. The Receiver operator curve (ROC) with the calculation of area under the curve (AUC) was used to evaluate the predictive ability of outcome events within 1-, 2- and 3-year. All tests were conducted on a two-sided, and *P* value < 0.05 were considered statistically significant.

## Results

### miR-182 promoter methylation is higher in elderly and unfit AML samples than in normal controls (NCs)

**Fig. 1:**
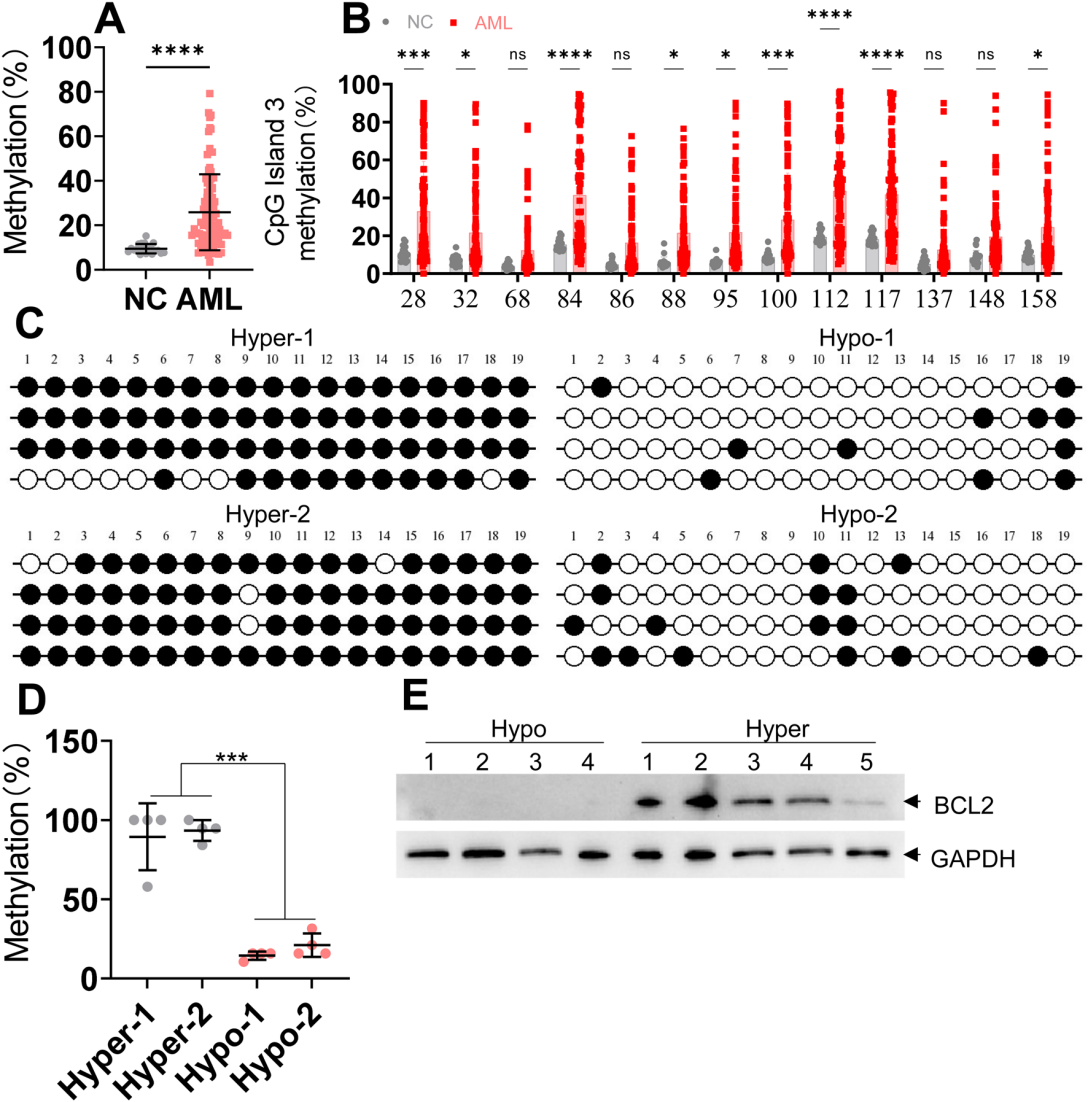
miR-182 promoter methylation frequency is higher in elderly or unfit AML patients than in NC samples. **A** MethylTarget™ assay was performed to analyze DNA methylation percentage of CpG islands 3 in 94 elderly or unfit AML patients and 20 NC samples. **B** The methylation frequency of individual CpG sites by MethylTarget™ assay was analyzed in 94 elderly or unfit AML patients and 20 NC samples. **C** and **D** Bisulfite-genomic sequencing was used to assess the methylation frequency in two AML patients with miR-182 promoter hypermethylation and two AML patients with miR-182 promoter hypomethylation. Four colonies were shown for each AML sample. Each row of the circle represents an individual clone. Empty and black circles represent unmethylated and methylated CpG dinucleotides, respectively (C). The statistical analysis of methylation frequency is shown (D). **E** BCL2 protein expression was measured in four AML samples with miR-182 promoter hypomethylation and five AML samples with hypermethylation. *P < 0.05; ***P < 0.001; ****P < 0.0001. ns: Not significant

Our reports have demonstrated that a total of three CpG islands (CpG island 1–3) are located 6–12 kb upstream of miR-182, and the methylation frequency is higher in CpG island 3 than that in CpG island 2 and 1 in leukemic cells [15, 16]. CpG island 3, representing the actual methylation frequency, was selected for the following analyses (Figure S1A and B). DNA was extracted from BM mononuclear cells of 94 elderly and unfit AML (Table S1) at initial diagnosis and 20 NC samples to explore miR-182 promoter methylation frequency. The average methylation frequency of CpG island 3 at the miR-182 promoter was 25.9% in 94 AML samples, and this methylation frequency was significantly higher in AML than that in 20 NC samples (9.4%) by MethylTarget™ assays (*P* < 0.0001, Figs. 1A, S2A–B, and S3A–B). Because MethylTarget™ assays include 13 CpG sites, we subsequently analyzed the methylation frequency of individual CpG sites. 9 of 13 CpG sites had higher methylation frequency in AML than those in NC samples (Fig. 1B). The detailed 13 CpG sites for MethylTarget™ assays were indicated in Figure S4A.

Our results from MethylTarget™ assays demonstrated that most AML patients present relative hypermethylation at initial diagnosis, although 11.7% of AML patients exhibited hypomethylation at the miR-182 promoter (< 10%). To further validate the methylation frequency of CpG island 3, bisulfite genomic sequencing was performed in two AML samples with miR-182 promoter hypermethylation and two with hypomethylation, which were categorized based on the results of MethylTarget™ assays. Consistent with MethylTarget™ assays, methylation frequency is substantially higher in two AML samples with miR-182 promoter hypermethylation than that in two AML with miR-182 promoter hypomethylation (Fig. 1C and D). The detailed 19 CpG sites for bisulfite genomic sequencing were indicated in Figure S4B.

As reported, AML patients with high BCL2 protein expression are sensitive to VEN + HMA treatment in vitro and in vivo [9, 20, 21]. Lost expression of BCL2 protein is considered the main factor leading to resistance to VEN + HMA treatment in AML patients [22]. We then determined whether AML cells with miR-182 promoter hypermethylation were associated with high BCL2 protein levels. We measured BCL2 protein expression in five AML samples with miR-182 promoter hypermethylation and four with hypomethylation. As expected, AML samples with miR-182 promoter hypermethylation had higher BCL2 protein expression compared with those with hypomethylation (Fig. 1E).

### AML cells with miR-182 promoter hypermethylation are more sensitive to VEN treatment in vitro and in vivo

**Fig. 2:**
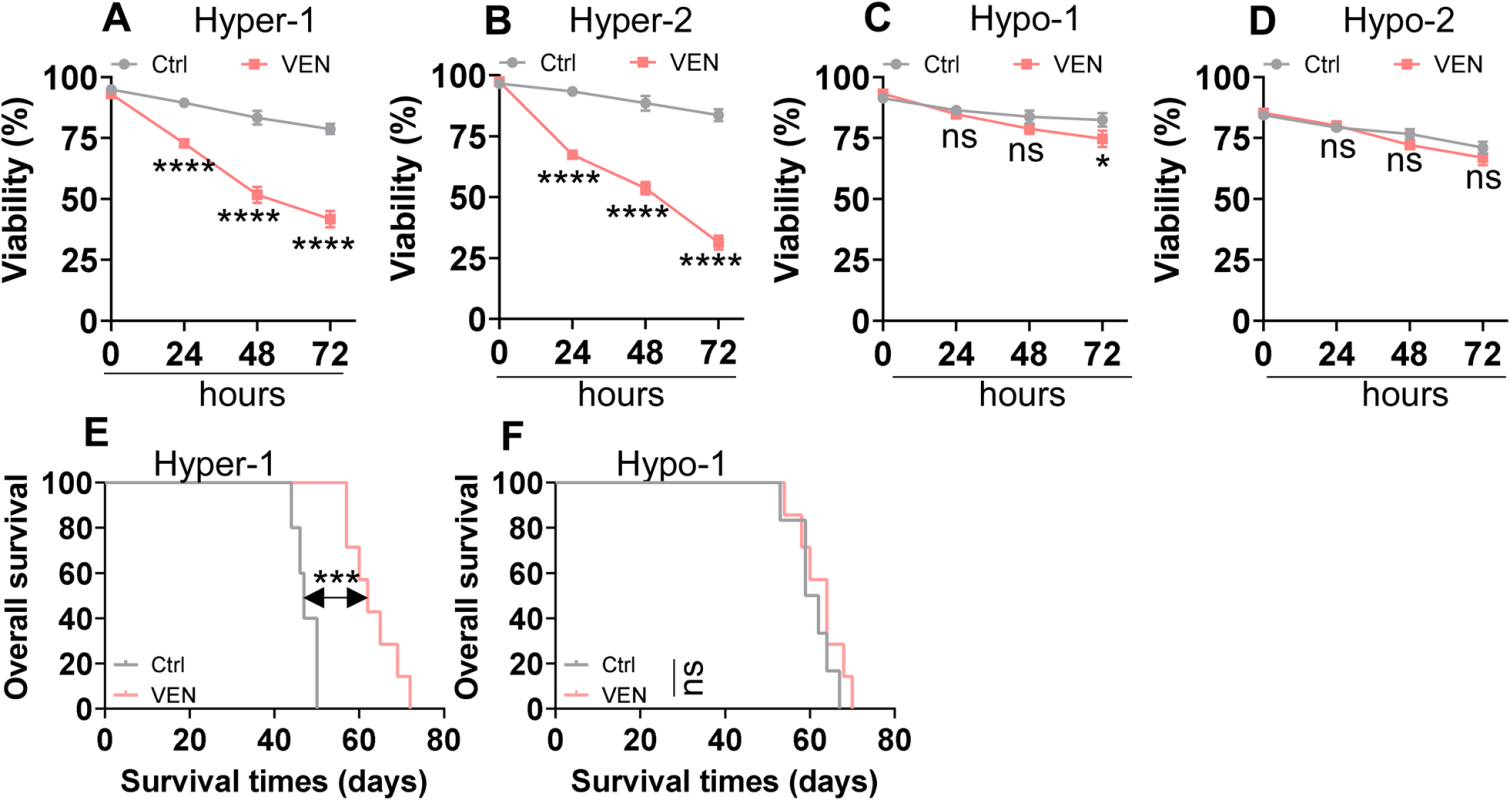
AML cells with miR-182 promoter hypermethylation are more sensitive to VEN treatment in vitro and in vivo. **A**–**D** Cell viability was measured in two AML cells with miR-182 promoter hypermethylation and two AML cells with hypomethylation, which were treated with or without VEN (0.5 μM) for 24 and 48 h. **E** and **F** OS was measured in NSG mice xenografted with one AML cells with miR-182 promoter hypermethylation and one AML cells with hypomethylation treated with or without VEN. *P < 0.05; ***P < 0.001; ****P < 0.0001. ns: Not significant

To further assess and compare the effects of VEN treatment in primary AML with miR-182 promoter hypermethylation or hypomethylation, AML with miR-182 promoter hypermethylation-1 and -2 and AML with miR-182 promoter hypomethylation-1 and -2 were treated with VEN (0.5 μM) at 24, 48, and 72 h, and viability was measured in vitro. VEN treatment significantly inhibited viability in AML cells with miR-182 promoter hypermethylation-1 and -2 (Fig. 2A and B) but did not markedly decrease viability in AML cells with miR-182 promoter hypomethylation-1 and -2 (Fig. 2C and D). Furthermore, we xenografted AML cells with miR-182 promoter hypermethylation-1 and AML with miR-182 promoter hypomethylation-1 in NSG mice and treated with or without VEN in vivo. VEN treatment substantially extended the OS in hyper-1-xenografted mice (Fig. 2E). However, VEN treatment did not affect the OS in hypo-1-xenografted mice (Fig. 2F). These results preliminarily demonstrated that AML with miR-182 promoter hypermethylation might be more sensitive to VEN treatment than AML with miR-182 promoter hypomethylation.

### No significant difference in baseline characteristics between AML patients with miR-182 promoter hypermethylation and hypomethylation

Ninety-four unfit newly diagnosed patients treated with AZA + VEN for at least one cycle were enrolled in this study (Table S1). The median age at diagnosis was 69 years, and 25 patients (26.6%) were older than 75 years. 42 AML patients (44.7%) were 3 or 4 according to the Eastern Cooperative Oncology Group (ECOG). For 2024 European LeukemiaNet (ELN) risk stratification [23], 44 AML patients (46.8%) were in the adverse risk group. We next analyzed patient characteristics, including main demographic and clinical and laboratory features, in AML patients with miR-182 promoter hypomethylation and hypermethylation. There were no statistically significant differences between the two groups’ baseline characteristics (Table S1).

### The average time to obtain CR/CRi in AML patients with miR-182 promoter hypermethylation is shorter than that in AML patients with miR-182 promoter hypomethylation

**Fig. 3:**
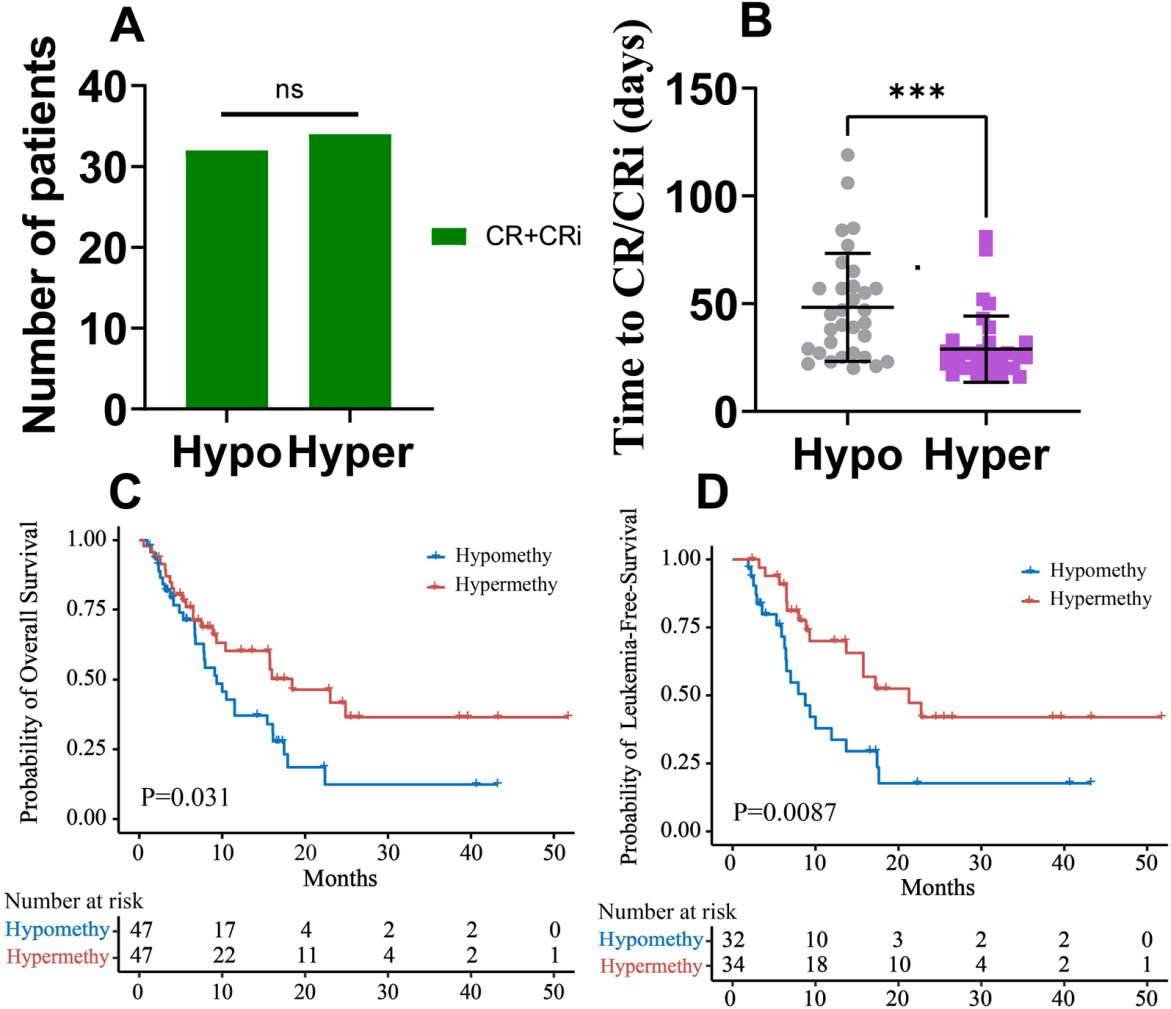
Evaluation of AZA + VEN treatment efficiency in AML patients with miR-182 promoter hypermethylation and hypomethylation. AML patients were divided into hypermethylation (> 20.21%) and hypomethylation (< 20.21%) according to median value. **A** The complete remission (CR)/CR with incomplete hematologic recovery (CRi) response numbers were calculated in AML patients with miR-182 promoter hypermethylation and hypomethylation. **B** The average time to achieve CR/CRi was analyzed in AML patients with miR-182 promoter hypermethylation and hypomethylation. **C** and **D** The impact of miR-182 promoter hypermethylation and hypomethylation on overall survival (OS, C) and leukemia-free survival (LFS, D) among AML patients. ***P < 0.001; ns: Not significant

We first assessed the CR/CRi status in AML patients with miR-182 promoter hypomethylation and hypermethylation. A total of 32 patients (68.1%) achieved CR/CRi in AML patients with miR-182 promoter hypomethylation (Fig. 3A), while the CR/CRi was 72.3% in AML patients with miR-182 promoter hypermethylation (Fig. 3A). There was no statistically significance of CR/CRi rate observed in AML patients with miR-182 promoter hypermethylation and hypomethylation by Fisher’s exact test (Fig. 3A). However, the average time to obtain CR/CRi was significantly shorter in AML patients with miR-182 promoter hypermethylation (28.9 days) than that in AML patients with hypomethylation (48.3 days) (*P* = 0.0003, Fig. 3B).

### miR-182 promoter methylation is a prognostic biomarker for OS and LFS

We subsequently analyzed the OS and LFS in 94 AML patients. The median OS was significantly shorter in AML patients with miR-182 promoter hypomethylation (9.33 months, 95% CI 6.77–16.13) compared with those with miR-182 promoter hypermethylation (18.43 months, 95% CI 10.4–NA) (*P* = 0.031, Fig. 3C). In addition, the median LFS was also shorter in AML patients with miR-182 promoter hypomethylation (8.77 months, 95% CI 6.43–17.63) than in those with miR-182 promoter hypermethylation (21.27 months, 95% CI 15.77–NA) (*P* = 0.0087, Fig. 3D). We further assessed the number of relapsed AML patients in both groups. Fifteen AML patients with miR-182 promoter hypomethylation (31.9%) recurred, but nine AML patients with miR-182 promoter hypermethylation (19.1%) relapsed (*P* = 0.156).

To determine potential factors affecting OS and LFS, univariable survival analyses were conducted by Cox analyses (Table 1). The univariable analyses revealed that miR-182 promoter methylation status as a categorical variable at diagnosis had a significant impact on OS and LFS (OS: HR 0.371 95% CI 0.210–0.657, *P* < 0.001; LFS: HR 0.293, 95% CI 0.149–0.579, *P* < 0.001). In addition, the following clinical characteristics, including BM blast, ELN risk group, and *TP53* mutation, were significantly associated with OS and LFS (Table 1). Additionally, variables such as achieved CR/CRi and the mutations in *STAG2* and *NPM1* correlated with OS but not with LFS (Table 1). Subsequently, we enrolled variables with statistical differences in univariable analyses (*P* < 0.05) into the multivariable analyses. As indicated in Table 2, in addition to the non-adverse ELN risk group and achieved CR/CRi, miR-182 promoter hypermethylation at diagnosis was a novel independent predictor for longer OS (HR 0.512, 95% CI 0.274–0.957, *P* = 0.036) and LFS (HR 0.358, 95% CI 0.175–0.733, *P* = 0.005).

**Table 1:** Univariable analysis of clinical factors for the survival in AML patients

	Overall survival HR (95% CI)	*P*-value	Leukemia-free survival HR (95% CI)	*P*-value
Age > 60 vs. ≤ 60	0.861 (0.442–1.677)	0.660	0.938 (0.411–2.138)	0.878
Sex M vs. F	1.124 (0.649–1.946)	0.678	1.432 (0.728–2.816)	0.298
WBC > 20 vs. ≤ 20	0.958 (0.556–1.653)	0.878	1.233 (0.644–2.356)	0.873
Platelets > 50 vs. ≤ 50	0.828 (0.480–1.430)	0.499	1.115 (0.581–2.141)	0.743
ECOG 3–4 vs.0–2	0.969 (0.562–1.670)	0.910	0.875 (0.458–1.673)	0.687
Bone marrow blast > 60 vs. ≤ 60	0.485 (0.276–0.850)	**0.011**	0.490 (0.251–0.958)	**0.037**
ELN risk group adverse vs non-adverse	3.473 (1.971–6.117)	** < 0.001**	2.731 (1.386–5.382)	**0.004**
Achieved CR/CRi	0.184 (0.102–0.331)	** < 0.001**		
Methylation level hypermethylation vs. hypomethylation	0.371 (0.210–0.657)	** < 0.001**	0.293 (0.149–0.579)	** < 0.001**
DNMT3A	0.870 (0.409–1.850)	0.717	1.067 (0.468–2.433)	0.877
IDH1	0.786 (0.283–2.183)	0.644	0.812 (0.286–2.300)	0.695
IDH2	0.607 (0.323–1.141)	0.121	0.623 (0.300–1.294)	0.204
JAK2	0.735 (0.101–5.334)	0.760	0.886 (0.121–6.493)	0.905
TP53	3.409 (1.776–6.544)	** < 0.001**	6.919 (2.333–20.517)	** < 0.001**
ASXL1	0.944 (0.443–2.010)	0.880	0.798 (0.332–1.915)	0.613
FLT3-ITD	0.431 (0.171–1.085)	0.074	0.716 (0.298–1.719)	0.455
FLT3-TKD	0.747 (0.319–1.752)	0.503	1.382 (0.631–3.025)	0.419
NPM1	0.342 (0.145–0.806)	**0.014**	0.641 (0.301–1.366)	0.250
STAG2	3.643 (1.400–9.483)	**0.008**	2.862 (0.674–12.162)	0.154
TET2	1.008 (0.503–2.021)	0.981	1.401 (0.651–3.013)	0.389
NF1	0.931 (0.290–2.991)	0.904	0.436 (0.060–3.185)	0.413
SRSF2	1.626 (0.726–3.641)	0.237	1.921 (0.670–5.506)	0.224
KIT	0.478 (0.066–3.467)	0.465	1.707 (0.523–5.572)	0.376
CEBPA	0.580 (0.180–1.867)	0.361	1.187 (0.462–3.050)	0.723
CEBPA-bZIP	0.446 (0.108–1.846)	0.265	0.483 (0.116–2.018)	0.318
PTPN11	1.280 (0.310–5.282)	0.733	1.420 (0.340–5.930)	0.630
RUNX1	1.361 (0.680–2.723)	0.384	1.502 (0.653–3.455)	0.338
K/NRAS	1.186 (0.577–2.435)	0.643	1.466 (0.690–3.111)	0.320
EZH2	0.295 (0.041–2.140)	0.227	0.330 (0.045–2.412)	0.275
U2AF1	4.041 (0.945–17.291)	0.060	2.223 (0.292–16.942)	0.441
BCOR	0.764 (0.238–2.456)	0.651	0.728 (0.175–3.028)	0.662

Bold values indicate that *P*-value is less than 0.05, suggesting that it is significant difference

**Table 2:** Multivariable analysis of clinical factors for survival in AML patients

	Overall survival HR (95% CI)	*P*-value	Leukemia-free survival HR (95% CI)	*P*-value
Bone marrow blast > 60 vs. ≤ 60	0.610 (0.314–1.187)	0.145	0.650 (0.318–1.327)	0.237
ELN risk group adverse vs. non-adverse	2.191 (1.128–4.258)	**0.021**	2.197 (1.091–4.422)	**0.027**
Achieved CR/CRi	0.314 (0.152–0.651)	**0.002**		
Methylation level hypermethylation vs. hypomethylation	0.512 (0.274–0.957)	**0.036**	0.358 (0.175–0.733)	**0.005**
TP53	0.988 (0.419–2.330)	0.977	3.020 (0.984–9.269)	0.053
STAG2	2.235 (0.715–6.987)	0.167		
NPM1	0.747 (0.280–1.995)	0.560		

Bold values indicate that *P*-value is less than 0.05, suggesting that it is significant difference

When miR-182 promoter methylation level at diagnosis was enrolled in the univariable Cox progression analyses as a continuous variable, it still remained positively associated with OS and LFS (OS: HR 0.972, 95% CI 0.954–0.992, *P* = 0.005; LFS: HR 0.962, 95% CI 0.939–0.987, *P* = 0.003), as displayed in Table S2. In addition, when enrolled into the multivariable analyses as a continuous variable, it kept independent effects on OS (HR 0.968, 95% CI 0.947–0.990, *P* = 0.004) and LFS (HR 0.969, 95% CI 0.946–0.991, *P* = 0.007) as well (Table S3). In other words, a 3.2% reduction in mortality was significantly associated with each percent of the miR-182 promoter methylation frequency at diagnosis. Concurrently, for every percentage rise in miR-182 promoter methylation frequency at diagnosis, there was a 3.1% reduction in recurrence or mortality rate.

We then explored whether the percentage of AML patients that receive allogenic hematopoietic stem cell transplantation (allo-HSCT) affects the OS and LFS in the hypermethylated and hypomethylated groups. Three AML patients with miR-182 protomer hypomethylation (6.4%) received allo-HSCT, and two AML patients with miR-182 protomer hypermethylation (4.3%) received allo-HSCT. The percentage of AML patients receiving allo-HSCT was very low and similar in both groups. Therefore, this is not the confounding factor for survival between the two groups.

### Predictive accuracy of the miR-182 promoter methylation in AML mortality risk via ROC curve

**Fig. 4:**
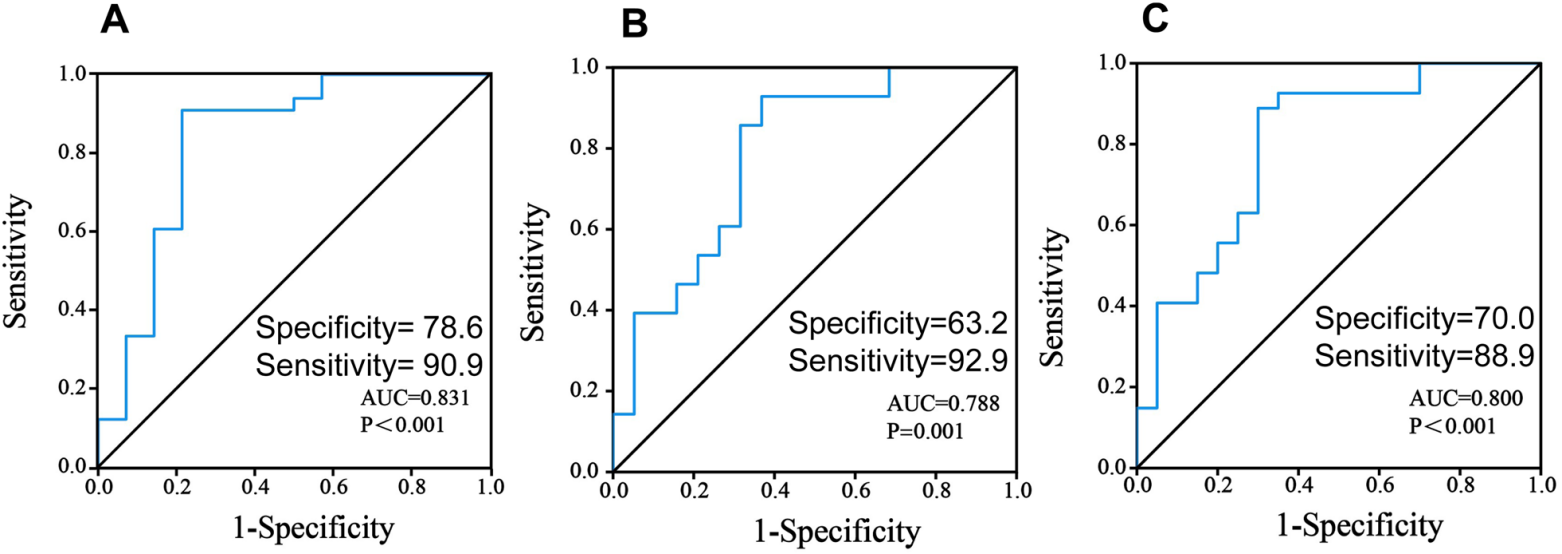
The area under the curve (AUC) analysis in AZA + VEN-treated AML patients. **A**–**C** AUC analysis of miR-182 promoter methylation for 1-year mortality (A), 2-year mortality (B), and 3-year mortality (C) was performed in AZA + VEN-treated AML patients

To determine the accuracy of the miR-182 promoter methylation level at diagnosis in predicting mortality risk in AML patients, we performed ROC curve analyses. The area AUC value was 0.831 for mortality at 1-year (95% CI 0.681–0.981, *P* < 0.001, Fig. 4A), 0.788 at 2-years (95% CI 0.649–0.926, *P* = 0.001, Fig. 4B), and 0.800 at 3-years (95% CI 0.667–0.933, *P* < 0.001, Fig. 4C). Therefore, our results demonstrated that the methylation frequency at diagnosis has a particular predictive effect on the prognosis of AZA + VEN-treated AML.

### Different methylation frequencies at different disease periods in AML patients

**Fig. 5:**
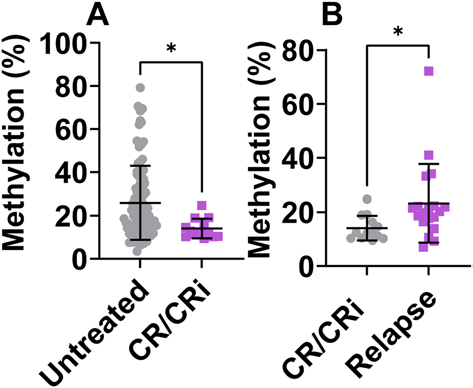
Different methylation frequencies in AML patients at diagnosis, with CR/CRi, and in relapse. **A** Methylation frequency at the miR-182 promoter was measured in AML patients at diagnosis and in AML patients achieving CR/CRi. **B** Methylation frequency at the miR-182 promoter was measured in AML patients achieving CR/CRi and in relapsed AML patients. *P < 0.05

We subsequently measured the frequency of miR-182 promoter methylation in BM cells obtained from AML patients who had undergone AZA + VEN treatment, across different time points. The average methylation frequency of miR-182 promoter was substantially lower in AML patients achieving CR/CRi than in newly diagnosed AML patients (14.1% vs 25.9%, *P* = 0.024, Fig. 5A). In addition, the average methylation level was substantially higher in relapsed AML patients than in AML patients achieving CR/CRi (22.6% vs 14.1%, *P* = 0.042, Fig. 5B).

### Leukemic differentiation stage does not affect miR-182 promoter methylation status in AML cells

**Fig. 6:**
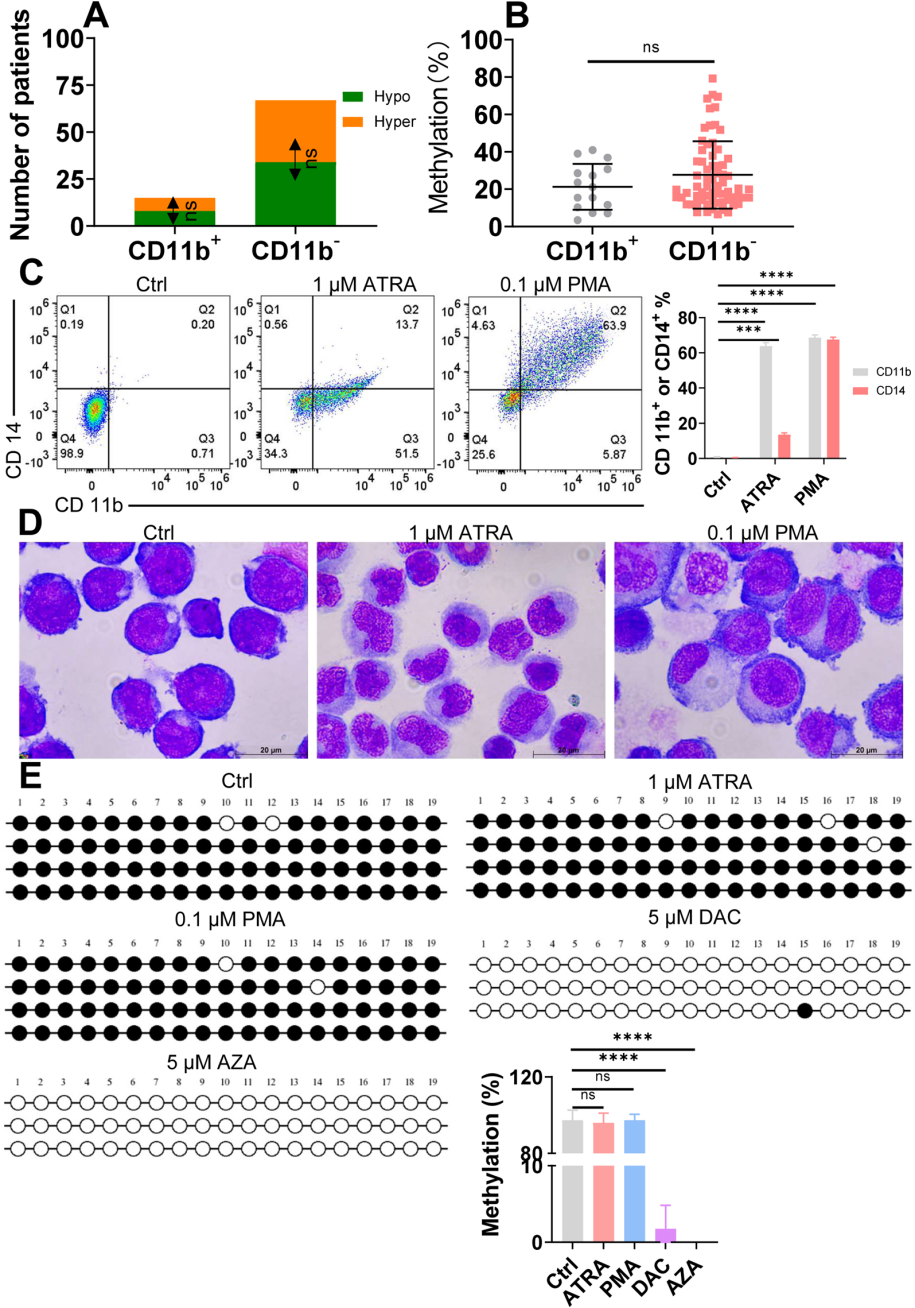
Leukemic differentiation stage does not affect miR-182 promoter methylation status. **A** 94 AML samples were divided in CD11b^+^ or CD11b^−^ cells. The frequencies of AML cells with miR-182 promoter hypermethylation and AML cells with hypomethylation were analyzed in CD11b^+^ or CD11b^−^ AML cells. **B** miR-182 promoter methylation frequency was analyzed in CD11b^+^ or CD11b^−^ AML cells. **C** CD11b and CD14 staining were performed by flow cytometer in U937 cells treated with 1 μM ATRA, 0.1 μM PMA, or DMSO (1:1000) as control (Ctrl) for 72 h. The representative plots (left) and statistical analysis of CD11b^+^ or CD14^+^ cells were shown (right). **D** Wright‒Giemsa staining was performed in U937 cells treated with 1 μM ATRA, 0.1 μM PMA, or Ctrl for 72 h. **E** Bisulfite-genomic sequencing was used to assess the methylation frequency of miR-182 promoter in U937 cells treated with 1 μM ATRA, 0.1 μM PMA, 5 μM DAC, 5 μM AZA, or Ctrl for 72 h. Each row of the circle represents an individual clone. Empty and black circles represent unmethylated and methylated CpG dinucleotides, respectively. The statistical analysis of methylation frequency is shown. ***P < 0.001; ****P < 0.0001. ns: Not significant

Although the cellular differentiation block is a fundamental characteristic of AML cells, AML cells have limited signs of differentiation [24]. Pei et al. report that AML cells in more differentiation stages, especially monocytic AML, resist VEN-based therapy [9]. Therefore, we subsequently explored whether miR-182 promoter methylation is associated with the differentiation stage. CD11b levels representing more differentiation stages were analyzed in 94 AML samples by flow cytometry. 15 of 94 (15.9%) AML samples are CD11b^+^ (Fig. 6A). 8 of 15 (53.3%) CD11b^+^ AML samples presented miR-182 promoter hypermethylation, and 7 of 15 (46.7%) CD11b^+^ AML samples presented hypomethylation (Fig. 6A). Therefore, AML with miR-182 promoter hypermethylation had an equal frequency of CD11b^+^ cells compared with AML with miR-182 promoter hypomethylation (Fig. 6A). Also, AML with miR-182 promoter hypermethylation had an equal frequency of CD11b^−^ cells compared with AML with miR-182 promoter hypomethylation (Fig. 6A). Furthermore, the frequency of miR-182 promoter methylation was similar in AML samples with CD11b^+^ compared with those with CD11b^−^ (Fig. 6B).

We next determined whether AML cells in more differentiation stages affected miR-182 promoter methylation, which was measured in U937 cells treated with all-*trans* retinoic acid (ATRA) and phorbol 12-myristate 13-ac-etate (PMA) as differentiation inducers. CD14/CD11b staining and Wright‒Giemsa staining demonstrated that U937 cells successfully differentiated into more mature cells (Fig. 6C and D). ATRA and PMA treatments did not affect miR-182 promoter methylation frequency in U937 cells by bisulfite genomic sequencing (Fig. 6E). In contrast, DAC and AZA treatments as positive controls significantly decreased miR-182 promoter methylation frequency [16] (Fig. 6E). These results demonstrated that differentiation could not affect miR-182 promoter methylation in AML cells.

## Discussion

Although several studies have reported predictive biomarkers for the sensitivity of VEN-based regimens combined with HMAs for AML patients in clinics [25, 26], these predictive biomarkers for VEN-based treatment were not wholly determined in a real-world setting. For example, Intermediate-risk cytogenetics or *RUNX1* mutation predicts more favorable survival outcomes in VEN-based treatment for relapsed/refractory (R/R) AML patients [25]. In addition, myelomonocytic leukemia, upregulation of BCL2A1 and CLEC7A, as well as mutations of *PTPN11* and *KRAS*, confer resistance to VEN-based therapy [27]. More recently, Dohner et al*.* have reported that *TP53*, *FLT3-ITD*, *NRAS*, and *KRAS* mutation status are associated with three different OS [28]. However, these reports lack quantitative methods to accurately and rapidly assess the sensitivity to AZA + VEN treatment. For example, mutation sites of *PTPN11* and *KRAS* are not fixed and are difficult to rapidly and quantitatively measure. Also, the combination modes of *TP53*, *FLT3-ITD*, *NRAS*, and *KRAS* mutations are complexed and difficult to analyze. By contrast, MethylTarget™ assay provides a high throughput technique to measure the miR-182 promoter methylation rapidly. Additionally, the fairly good sensitivity, specificity, and clinical relevance of miR-182 promoter methylation discovered in our study make it a valuable tool for identifying and monitoring methylation changes associated with prognosis. Therefore, we offer a rapid and quantitative method to predict the clinical outcome in AZA + VEN-treated elderly or unfit AML patients by measuring miR-182 promoter methylation.

BCL2 plays a vital role in the survival of leukemic cells, especially for LSC, which has aberrantly high levels of BCL2 for survival [3]. Moreover, high expressions of BCL2 are associated with chemoresistance and adverse clinical outcomes in AML patients [29, 30]. Correspondingly, AML cells with high protein levels of BCL2 are commonly sensitive to VEN treatment [31]. By contrast, AML cells with low expressions of BCL2 or AML cells that do not depend on BCL2 for survival are resistant to VEN treatment [12, 32]. For example, AML cells depending on alternative BCL2 family members such as BCL2L1 or MCL1 for survival are resistant to VEN treatment [9, 33]. Thus, AML cells with miR-182 promoter hypermethylation have higher expression of BCL2 protein than those with miR-182 promoter hypomethylation, which partially sheds light on the consequence that AML patients with miR-182 promoter hypermethylation have better OS and LFS than those with hypomethylation. In addition to regulating BCL2 protein expression by miR-182 [16] and miR-15a/16-1 [34], RNA-binding protein YBX1 can regulate BCL2 mRNA and protein levels in an m^6^A-dependent manner [35]. We could not exclude the potential effects of miR-15a/16-1 and YBX1 on BCL2 protein expression in AML patients with miR-182 promoter hypermethylation or hypomethylation. However, these reports do not explore the relationship between BCL2 protein expression and VEN resistance.

Although the positive correlation between high levels of BCL2 protein and the response to AZA + VEN cotreatment seems robust [31], it is difficult to rapidly and quantitatively measure BCL2 protein level in AML cells. Here, we found that methylation frequency at the miR-182 promoter indirectly reflects the BCL2 protein level and predicts the outcome in AZA + VEN-treated AML patients. In this manuscript, we used the MethylTarget™ assay [36], a high throughput technique, to measure the miR-182 promoter methylation, which was confirmed by bisulfite sequencing. MethylTargetTM assay is suitable and cost-effective for detecting and quantifying methylation in large samples because of its high throughput. Thus, this assay is ideally suited for retrospective studies involving large samples. However, it is not suitable for small sample sizes. Therefore, a probe-based methylation-specific PCR (MSP) is being developed to measure miR-182 promoter methylation level.

Although our results demonstrated that AZA + VEN-treated AML patients with miR-182 promoter hypermethylation had higher OS and LFS than those with hypomethylation, the CR/CRi rates were similar in these two groups. This might be caused by the relatively small AML samples enrolled. However, the average time to obtain CR/CRi was significantly shorter in AML with miR-182 promoter hypermethylation than in AML with miR-182 promoter hypomethylation. The shorter time required to obtain CR/CRi reflects a rapid reduction in leukemia burden and has implications for reducing exposure to side effects. Consequently, the attenuated side effects positively impact long-term survival in AML patients, especially elderly AML patients.

Our study demonstrated that patients achieving CR/CRi had low levels of miR-182 promoter methylation (Fig. 5A), probably because AML patients achieving CR/CRi have a high percentage of normal hematological cells and a low percentage of leukemic cells. Normal hematological cells present miR-182 promoter hypomethylation [15, 16]. Thus, a high percentage of normal hematological cells in AML patients obtaining CR/CRi results in lower levels of miR-182 promoter methylation. Conversely, relapsed patients have a high percentage of resistant leukemic cells. These leukemic cells have miR-182 promoter hypermethylation [15, 16]. Thus, a high percentage of leukemic cells in relapsed AML patients results in higher levels of miR-182 promoter methylation (Fig. 5B). This could imply that methylation status at the miR-182 promoter predicts treatment response and disease outcome. Higher methylation levels at the miR-182 promoter silence the expression of miR-182, leading to alterations in downstream signaling pathways that promote disease progression, which requires further validation through additional experimental studies.

Our results suggest that AML patients with miR-182 promoter hypermethylation exhibit a comparable distribution of patient characteristics to those with miR-182 promoter hypomethylation. For example, mutations in some essential genes, such as *TP53* [37] and *IDH1/2* [38, 39], are distributed equally between AML cells with miR-182 promoter hypermethylation and hypomethylation (Table S1). Mutant IDH produces 2-hydroxyglutarate (2HG), which induces DNA hypermethylation [39, 40]. However, whether mutant IDH can regulate miR-182 promoter methylation is unknown. Therefore, miR-182 promoter methylation impacts the outcome in AZA + VEN-treated AML patients, probably independent of these patient characteristics. However, the relatively limited size of AML samples and the low mutation frequency might cause a distortion in the distribution.

Monocytic subpopulations representing more differentiation stages resist AZA + VEN treatment because monocytic subpopulations have low expression of BCL2 protein [9]. Our results demonstrated that there is no statistical difference in the frequency of miR-182 promoter methylation in AML with more differentiation stages than in immature stages. Moreover, ATRA and PMA induced differentiation [41] but did not affect miR-182 promoter methylation in U937 cells in vitro. Therefore, miR-182 promoter hypomethylation-mediated resistance of AZA + VEN treatment is independent of leukemic differentiation level. Although monocytic cells are more resistant to AZA + VEN treatment [9], no robust evidence has demonstrated that all monocytic FAB-M4/M5 patients are resistant to AZA + VEN treatment. It is probable that monocytic FAB-M4/M5 patients are heterogeneous concerning cytogenetic and molecular genetic abnormalities [24]. Our study also demonstrated that M1, M2, M4, and M5 patients were distributed equally between miR-182 promoter hypermethylation and hypomethylation. These results confirm that differentiation can not affect miR-182 promoter methylation.

## Conclusions

Here, our studies find the predictive biomarker of miR-182 promoter methylation for elderly or unfit AML treated with AZA + VEN, with fairly good sensitivity, specificity, and clinical relevance. miR-182 promoter hypermethylation predicts a better outcome in AZA + VEN-treated elderly or unfit AML patients. Additionally, methylation levels at the miR-182 promoter were associated with different disease periods in AML patients, such as initial diagnosis, CR/CRi and relapse. We are preparing a prospective study to assess the predictive outcome of miR-182 promoter methylation in elderly or unfit AML treated with AZA + VEN.

## Supplementary Information

Additional file 1.

Additional file 2.

Additional file 3.

Additional file 4.

Additional file 5.

Additional file 6.

Additional file 7.

Additional file 8.

Additional file 9.

## Data Availability

No datasets were generated or analysed during the current study.

## Abbreviations

Ven: Venetoclax
AZA: Azacitidine
DAC: Decitabine
BM: Bone marrow
FBS: Fetal bovine serum
HMA: Hypomethylating agent
HSPC: Hematopoietic stem and progenitor cell
miRNA: MicroRNA
CpG: Cytosine-phosphate-guanine
CR: Complete remission
MNCs: Mononuclear cells
GFP: Green fluorescent protein
OS: Overall survival
AML: Acute myeloid leukemia
LFS: Leukemia-free survival
LSC: Leukemia stem cells
NSG: NOD/SCID‑IL2Rγ mice
ROC: Receiver operator curve
ELN: European LeukemiaNet
ECOG: Eastern Cooperative Oncology Group
