# Supplementary figures and images for: miR-182 promoter hypermethylation predicts the better outcome of AML patients treated with AZA + VEN in a real-world setting
Source: Clin Epigenetics. 2025 Feb 5;17:18. doi: 10.1186/s13148-025-01823-1 (PMC11800541; doi:10.1186/s13148-025-01823-1)

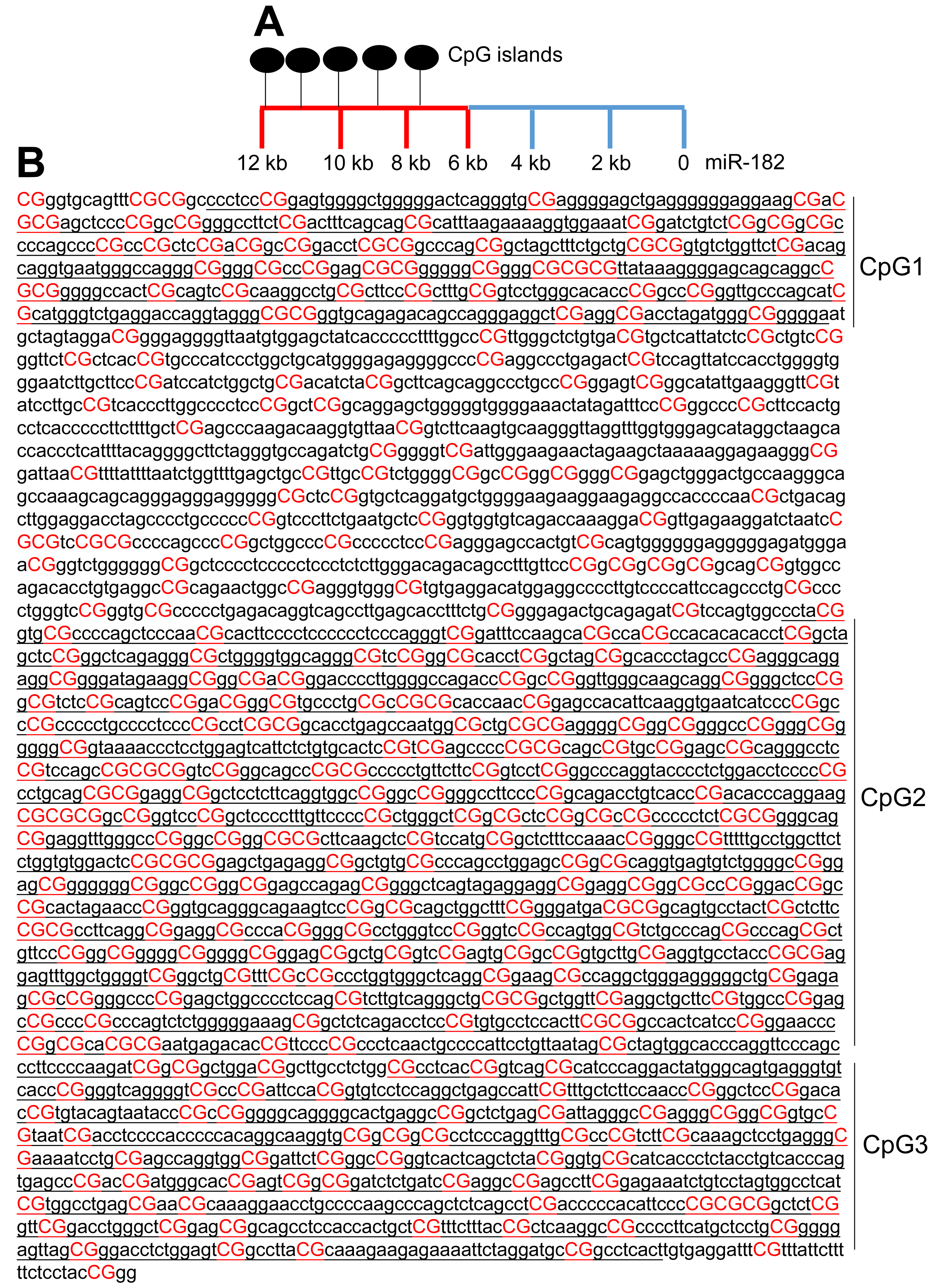

Supplement: Supplementary file 1 — Additional file 1. [file 13148_2025_1823_MOESM1_ESM.tif]

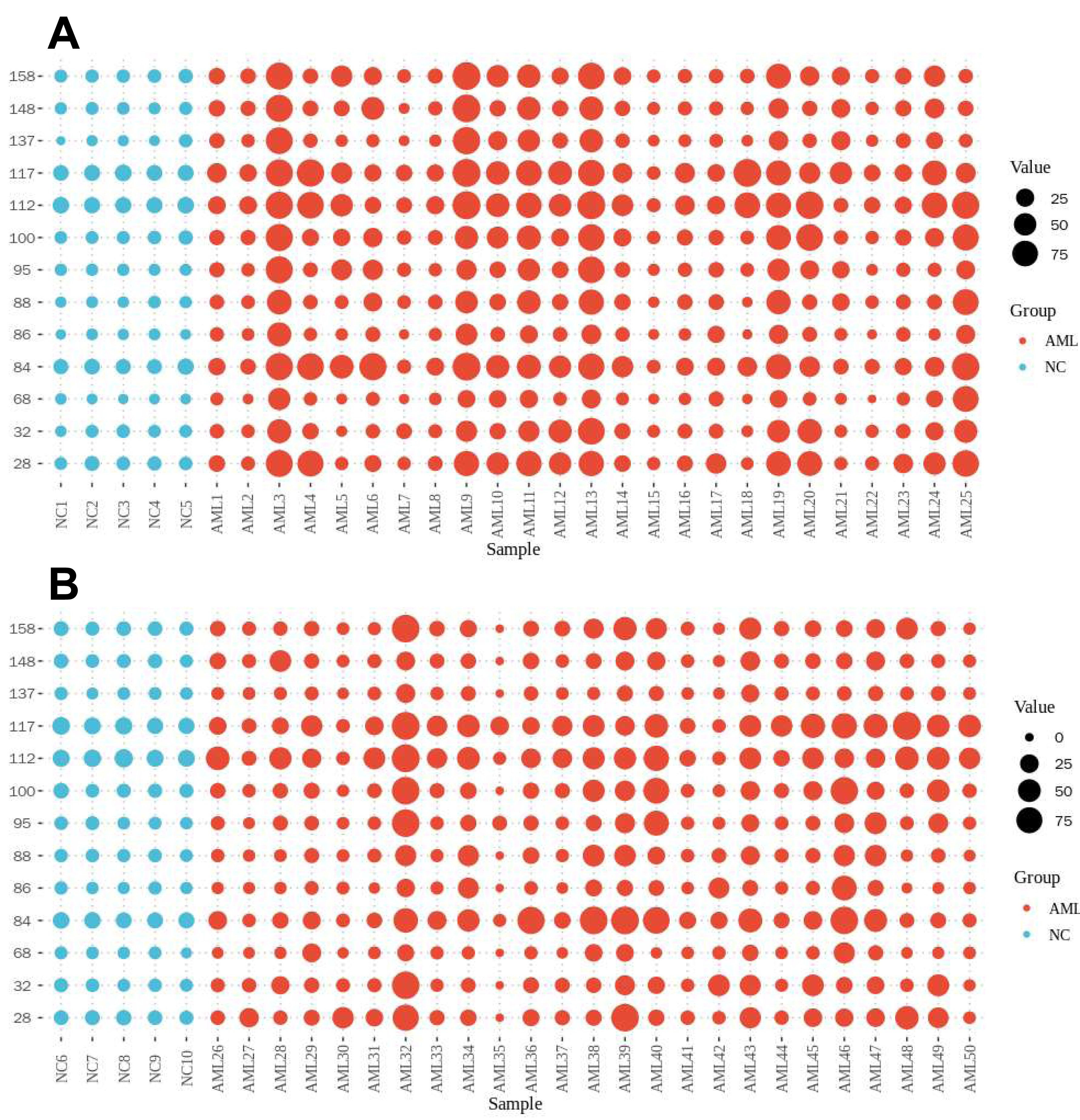

Supplement: Supplementary file 2 — Additional file 2. [file 13148_2025_1823_MOESM2_ESM.tif]

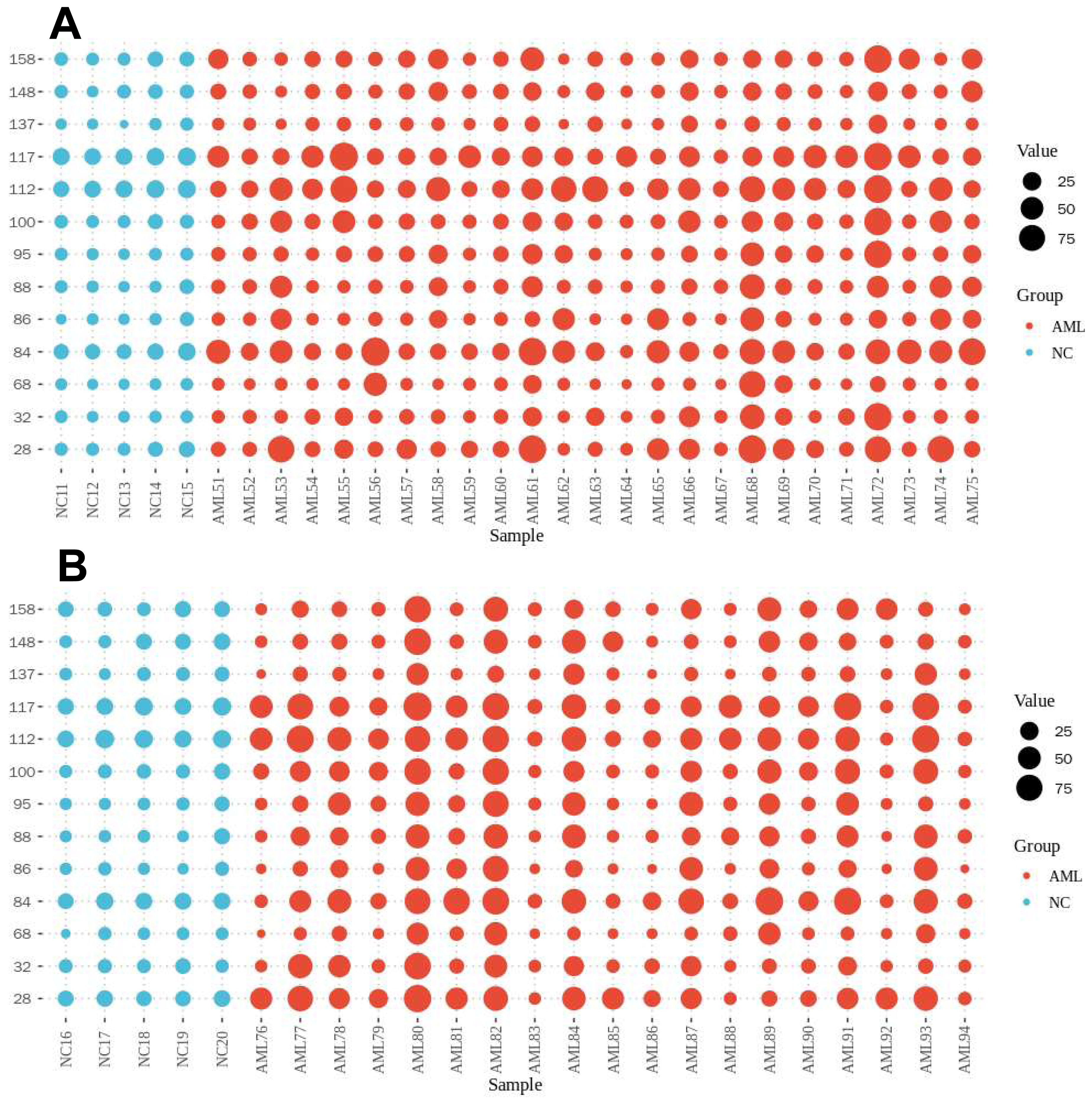

Supplement: Supplementary file 3 — Additional file 3. [file 13148_2025_1823_MOESM3_ESM.tif]

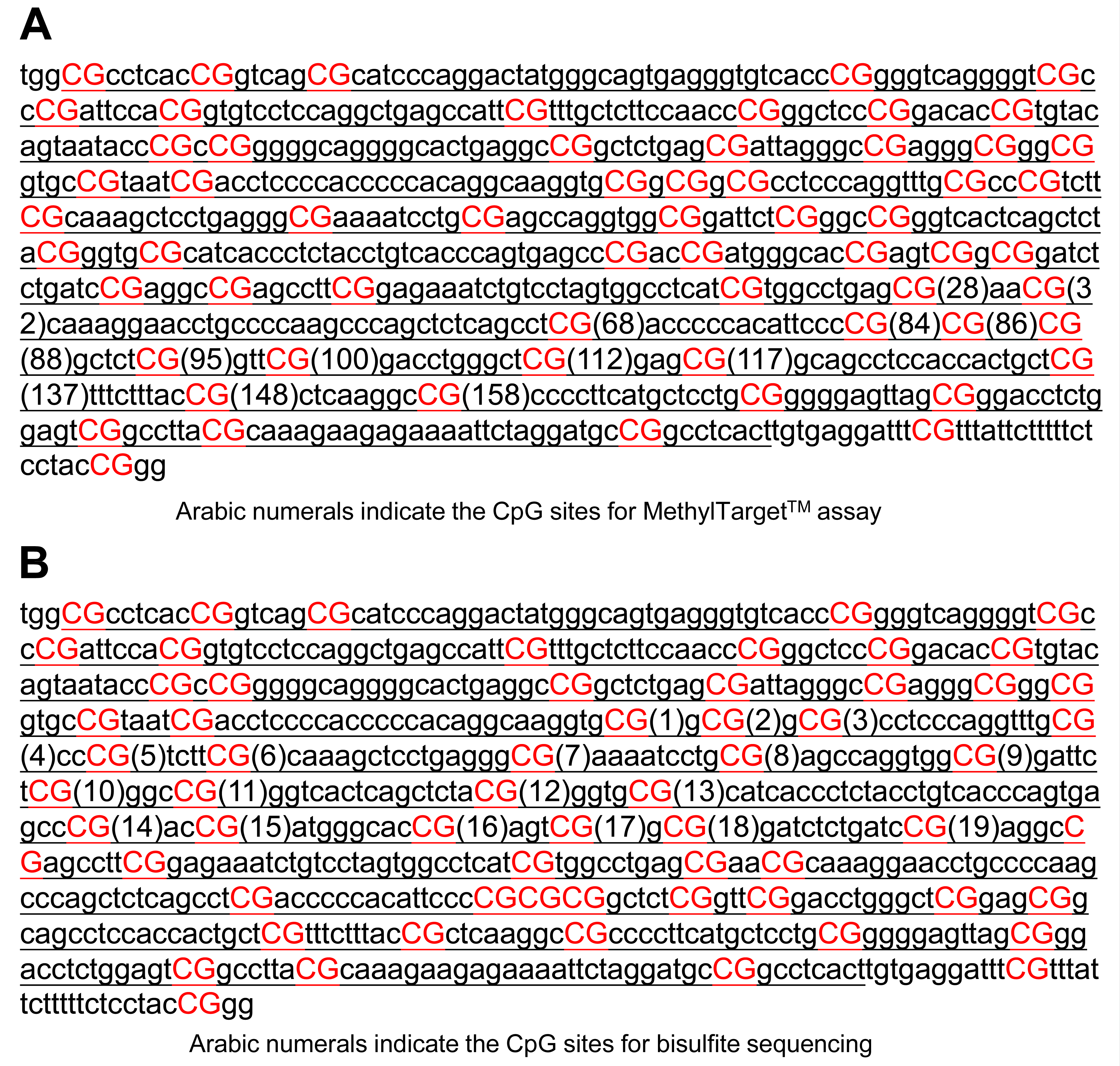

Supplement: Supplementary file 4 — Additional file 4. [file 13148_2025_1823_MOESM4_ESM.tif]
