# Supplementary material for: miR-182 promoter hypermethylation predicts the better outcome of AML patients treated with AZA + VEN in a real-world setting
Source: Clin Epigenetics. 2025 Feb 5;17:18. doi: 10.1186/s13148-025-01823-1 (PMC11800541; doi:10.1186/s13148-025-01823-1)
Supplement: Supplementary file 5 — Additional file 5. [file 13148_2025_1823_MOESM5_ESM.docx]

**Supplemental Figure Legend**

**Figure S1: The detailed distribution of three CpG islands at the miR-182 promoter.** (A) The regions indicating the CpG islands at the miR-182 promoter. (B) The detailed base information of three CpG islands.

**Figure S2: The methylation frequency of CpG island 3 at the miR-182 promoter by MethylTarget^TM^ assay.** (A and B) The detailed methylation of CpG island 3 in 10 NC (1-10) and 50 primary AML blasts (1-50).

**Figure S3:** **The methylation frequency of CpG island 3 at the miR-182 promoter by MethylTarget^TM^ assay.** (A and B) The detailed methylation of CpG island 3 in 10 NC (11-20) and 44 primary AML blasts (51-94).

**Figure S4: The individual CpG sites for MethylTarget^TM^ assay and bisulfite sequencing.** (A) 13 CpG sites for MethylTarget^TM^ assays were indicated by Arabic numerals, which mean the base number starting from the left primer. (B) 19 CpG sites for bisulfite genomic sequencing were indicated by Arabic numerals.
