# Supplementary material for: miR-182 promoter hypermethylation predicts the better outcome of AML patients treated with AZA + VEN in a real-world setting
Source: Clin Epigenetics. 2025 Feb 5;17:18. doi: 10.1186/s13148-025-01823-1 (PMC11800541; doi:10.1186/s13148-025-01823-1)
Supplement: Supplementary file 6 — Additional file 6. [file 13148_2025_1823_MOESM6_ESM.docx]

**Supplemental material and methods**

**Drugs and chemical reagents**

VEN (TargetMol, Boston, MA, USA), all-*trans* retinoic acid (ATRA, TargetMol), phorbol 12-myristate 13-ac-etate (PMA, TargetMol), 5'-Azacitidine (AZA, TargetMol), and decitabine (DAC, TargetMol) were dissolved in dimethyl sulphoxide (DMSO) and kept at -86 °C until use.

**Viability assay**

Viability was measured by the trypan blue exclusion test. AML cells treated with or without VEN (0.5 μM) for 24 and 48 h were mixed with trypan blue (0.1%, Thermo Scientific, Waltham, MA, USA), and cell viability was counted by a cell counter (VI-CELL XR, Beckman-Coulter, Brea, CA, USA).

**Wright‒Giemsa staining**

Leukemic cells were stained using standard protocols for morphological analysis[1]. In brief, leukemic cells were collected and centrifuged on slides by cytospin (Shandon, Runcorn, United Kingdom). Slides were stained by Wright‒Giemsa staining buffer for approximately 3-4 min after all slides were dried entirely. Cytospins were examined under a light microscope (Leica Microsystems Inc., Deerfield, IL, USA).

**CD11b and CD14 staining by ﬂow cytometry**

Differentiation antigens were measured by ﬂow cytometry (CytoFLEX LX, Beckman-Coulter, Brea, CA, USA). Brieﬂy, cells were collected, washed, and incubated with monoclonal anti-CD11b (BD Biosciences, San Jose, CA, USA) and anti-CD14 (BD Biosciences) for 30 min at room temperature. Non-specific IgG antibody was used as a negative control. Fluorescence intensity was analyzed by FlowJo software (Tree Star Ashland, OR, USA).

**Western blot**

Western blot analysis was performed using standard techniques. Briefly, primary AML cells were harvested and lysed by 1×SDS lysis buffer (Thermo Scientific, Waltham, MA, USA). Extracted protein was heated at 100℃ for 5 min and rapid cooling at 4℃ for 5 min three times. BCA assay (Thermo Scientific) was used to measure protein concentration. Proteins (30 μg/well) for every sample were fractionated by electrophoresis and were transferred to PVDF membranes (0.45 μm, Bio-Rad). After incubation with TBS solution with 5% skim milk powder for 2 h, PVDF blots were incubated with primary antibodies overnight at 4 °C, followed by incubation with a secondary HRP-conjugated antibody for 1 h. After blots were washed by TBST with 0.1% tween 20, signals were measured by chemiluminescence reagents (Bio-Rad) with an imaging system (Bio-Rad). The following antibodies were used: BCL2 (ab182858, Abcam). As necessary, blots were stripped and reprobed with GAPDH antibody (ab8245, Abcam) as an endogenous control.

**DNA methylation detection by MethylTarget^TM^ assay**

Genomic DNA was extracted from BM mononuclear cells of untreated AML patients and healthy volunteers as normal controls (NC) using the DNA Mini kit (QIAGEN, Hilden, Germany) according to the manufacturer's instructions. CpG islands at miR-182 promoter were selected according to the following criteria:1. >200 bp length; 2. cytosine and guanine content >50%; 3. ≥0.60 ratios of observed/expected dinucleotides CpG. Bisulfite conversion of all unmethylated cytosine to uracil was performed by the EZ DNA Methylation™-GOLD Kit (ZYMO RESEARCH, Irvine, CA, USA). We filtered out the samples with bisulfite conversion rate < 98% and used MethylTarget^TM^ assay (Genesky BioTech, Shanghai, China) as targeted bisulfite sequencing. miR-182 promoter methylation was measured in 94 untreated AML patients, 13 AML patients obtained CR/CRi, and 19 relapsed AML patients.

**Bisulfite sequencing**

DNA was extracted from BM mononuclear cells of AML patients. Then, the DNA was treated with sodium bisulfite and purified by the EZ DNA Methylation™-GOLD Kit (ZYMO RESEARCH, USA). Sodium bisulfite-treated DNA was used as the template to amplify the CpG island at the miR-182 promoter by PCR. The following sequences of primers were used: F: 5′-TTT TTT TAT TTT TAT AGG TAA GGT G-3′; R: 5′-TAA AAC CAC TAA AAC AAA TTT CTC C-3′. PCR products were subcloned into the pUC18 vector for direct sequencing.

**Treatment procedure**

The therapy protocol combined AZA with VEN, following a specific dosing regimen. Patients received VEN initially at a dose of 100 mg orally on day 1, whose dose ramped up to 200 mg on day 2, and then maintained at 400 mg orally once daily from day 3 to day 28 in the first course of treatment. Concurrently, AZA, at a dose of 75 mg/m^2^ of body surface area, is administered intravenously for seven consecutive days starting from day 1. Starting from the second course, patients received 400 mg of VEN daily from day 1 to day 28. When using voriconazole or posaconazole in combination, the VEN dose was adjusted to 100 mg orally per day. In the event of an uncontrollable active infection or severe organ damage, the therapeutic approach was adjusted in accordance with the 2017 recommendations issued by an international expert panel acting on behalf of the European LeukemiaNet (ELN)[2].

**Definition of unfit AML patients**

Patients, precluded intensive chemotherapy, had at least one of the following coexisting conditions: 1. A history of congestive heart failure that required treatment, or a history of myocardiopathy with an ejection fraction of 50% or less. 2. chronic stable angina. 3. a history of lung disease characterized by a reduced diffusing capacity of the lung for carbon monoxide, below 65%, or a forced expiratory volume in one second (FEV1) less than 65%. 4. an Eastern Cooperative Oncology Group (ECOG) performance-status score of 2 or 3.

**Response criteria and outcomes**

For early assessment of disease response to AZA+VEN cotreatment, BM aspirate was made on days 21 to 28 after the first circle according to National Comprehensive Cancer Network (NCCN) Guidelines insights[3]. The therapeutic response was classified as composite CR (including CR and CRi), PR, and NR, adhering to the 2017 recommendations issued by an international expert panel on behalf of the European LeukemiaNet (ELN)[2]. CR, represented the complete fulfillment of a set of rigorous criteria, which encompassed bone marrow blasts comprising less than 5% of the total, the absence of circulating blasts in the peripheral blood, the absence of extramedullary disease manifestations, an absolute neutrophil count (ANC) of at least 1,000/μL, and a platelet count of at least 100,000/μL. Similarly, CRi fulfilled all these CR criteria except for the presence of residual neutropenia with an ANC less than 1,000/μL or thrombocytopenia with a platelet count less than 100,000/μL, indicating that the patient had achieved most, but not all, of the necessary hematologic recovery. PR fulfills hematologic CR criteria but with a 5-25% bone marrow blast percentage, reduced by ≥50% from pretreatment levels. Patients who were eligible for response evaluation but did not meet the criteria for CR, CRi, or PR were categorized as having NR. The first endpoint of interest was death from any cause. OS was calculated from the first day of AZA+VEN treatment until death, loss of follow-up, or end of follow-up. Relapse or death was considered as the second endpoint of interest. Leukemia-free survival (LFS) was calculated from the date of CR to relapse or death, loss of follow-up, or end of follow-up. The date of the last follow-up is 31st May 2024.

**Engraftment of NOD/SCID‑IL2Rγ mice (NSG)**

Female 10-week-old NSG mice (Shanghai Model Organisms Center, Shanghai, China) were intraperitoneally injected with busulfan (30 mg/kg; Sigma) one day before transplantation[4]. A total of 2×10^6^ BM cells from AML patients were transplanted into NSG mice by vein injection. NSG mice were randomized into two groups and treated with vehicle or 100 mg VEN/kg body weight for seven consecutive days at about four weeks after transplantation. VEN was formulated in 90% polyethylene glycol 300 and 10% DMSO. OS time was determined from the first day of the experiment until death.

References

1. Song MG, Gao SM, Du KM, Xu M, Yu Y, Zhou YH, et al. Nanomolar concentration of NSC606985, a camptothecin analog, induces leukemic-cell apoptosis through protein kinase Cdelta-dependent mechanisms. Blood*.* 2005;105;9:3714-21.

2. Dohner H, Estey E, Grimwade D, Amadori S, Appelbaum FR, Buchner T, et al. Diagnosis and management of AML in adults: 2017 ELN recommendations from an international expert panel. Blood*.* 2017;129;4:424-47.

3. Pollyea DA, Bixby D, Perl A, Bhatt VR, Altman JK, Appelbaum FR, et al. NCCN Guidelines Insights: Acute Myeloid Leukemia, Version 2.2021. J Natl Compr Canc Netw*.* 2021;19;1:16-27.

4. Fang J, Liu X, Bolanos L, Barker B, Rigolino C, Cortelezzi A, et al. A calcium- and calpain-dependent pathway determines the response to lenalidomide in myelodysplastic syndromes. Nat Med*.* 2016;22;7:727-34.
