# Supplementary material for: miR-182 promoter hypermethylation predicts the better outcome of AML patients treated with AZA + VEN in a real-world setting
Source: Clin Epigenetics. 2025 Feb 5;17:18. doi: 10.1186/s13148-025-01823-1 (PMC11800541; doi:10.1186/s13148-025-01823-1)
Supplement: Supplementary file 7 — Additional file 7. [file 13148_2025_1823_MOESM7_ESM.docx]

Table S1. Baseline characteristics of enrolled AML patients

| Characteristic | Hypomethylation group (n = 47) | Hypermethylation group (n = 47) | p-value |
| --- | --- | --- | --- |
| Median age, years (range) | 68.0(55-84) | 70(54-91) | 0.442 |
| Male gender, no. (%)  Female gender, no. (%) | 23(48.9%) | 21(44.7%) | 0.679 |
|  | 24(51.1%) | 26(55.3%) |  |
| FAB subtypes, no. (%) |  | | 0.898 |
| M0/M1 | 4(8.5%) | 3(6.4%) |  |
| M2 | 11(23.4%) | 11(23.4%) |  |
| M4 | 10(21.3%) | 13(27.7%) |  |
| M5 | 22(46.8%) | 20(42.6%) |  |
| Median WBC, ×10^9^ /L (range) | 7.7(0.97-363.4) | 20.7(0.62-326.53) | 0.610 |
| Median platelets, ×10^9^ /L (range) | 65(13-582) | 47(3-303) | 0.156 |
| ECOG score, no. (%) |  | | 0.407 |
| 0-2  3-4 | 28(59.6%) | 24(51.1%） |  |
|  | 19(40.4%) | 23(48.9%) |  |
| Median bone marrow blast (range) | 51(7.5-98) | 67.5(8.5-93.5) | 0.146 |
| ELN risk group, n (%) |  | | 0.694 |
| Favorable | 7(14.9%) | 9(19.1%) |  |
| Intermediate | 16(34.0%) | 18(38.3%) |  |
| Adverse | 24(51.1%) | 20(42.6%) |  |
| Gene mutation type, n (%) |  | | |
| DNMT3A | 10(21.3%) | 4(8.5%) | 0.147 |
| IDH1 | 2(4.3%) | 1(2.1%) | 1.0 |
| IDH2 | 8(17.0%) | 12(25.5%) | 0.313 |
| JAK2 | 2(4.3%) | 0 | 0.475 |
| TP53 | 9(19.1%) | 9(19.1%) | 1.0 |
| ASXL1 | 11(23.4%) | 5(10.6%) | 0.1 |
| FLT3-ITD | 4(8.5%) | 9(19.1%) | 0.135 |
| FLT3-TKD | 6(12.8%) | 7(14.9%) | 0.765 |
| NPM1 | 8(17.0%) | 10(21.3%) | 0.600 |
| STAG2 | 2(4.3%) | 3(6.4%) | 1.0 |
| TET2 | 10(21.3%) | 6(12.8%) | 0.272 |
| CUX1 | 1(2.1%) | 0 | 1.0 |
| NF1 | 3(6.4%) | 3(6.4%) | 1.0 |
| SRSF2 | 6(12.8%) | 3(6.4%) | 0.483 |
| KIT | 1(2.1%) | 2(4.3%) | 0.554 |
| CEBPA | 3(6.4%) | 7(14.9%) | 0.181 |
| CEBPA-bZIP | 0 | 3(6.4%) | 0.241 |
| PTPN11 | 2(4.3%) | 1(2.1%) | 1.0 |
| RUNX1 | 9(19.1%) | 7(14.9%) | 0.583 |
| CBL | 1(2.1%) | 0 | 1.0 |
| NRAS | 5(10.6%) | 6(12.8%) | 0.748 |
| DDX41 | 1(2.1%) | 1(2.1%) | 1.0 |
| EZH2 | 1(2.1%) | 3(6.4%) | 0.609 |
| U2AF1 | 2(4.3%) | 1(2.1%) | 1.0 |
| BCOR | 5(10.6%) | 2(4.3%) | 0.432 |
| KRAS | 2(4.3%) | 3(6.4%) | 1.0 |
| PHF6 | 1(2.1%) | 2(4.3%) | 1.0 |
