# Supplementary material for: miR-182 promoter hypermethylation predicts the better outcome of AML patients treated with AZA + VEN in a real-world setting
Source: Clin Epigenetics. 2025 Feb 5;17:18. doi: 10.1186/s13148-025-01823-1 (PMC11800541; doi:10.1186/s13148-025-01823-1)
Supplement: Supplementary file 8 — Additional file 8. [file 13148_2025_1823_MOESM8_ESM.docx]

Table S2. Univariable analysis of clinical factors for the survival in AML patients

|  | Overall survival  HR (95% CI) | p-value | Leukemia-free survival  HR (95% CI) | p-value |
| --- | --- | --- | --- | --- |
| Age > 60 vs.≤ 60 | 0.861(0.442-1.677) | 0.660 | 0.938(0.411-2.138) | 0.878 |
| Sex M vs F | 1.124(0.649-1.946) | 0.678 | 1.432(0.728-2.816) | 0.298 |
| WBC > 20 vs.≤ 20 | 0.958(0.556-1.653) | 0.878 | 1.233(0.644-2.356) | 0.873 |
| Platelets >50 vs.≤ 50 | 0.828(0.480-1.430) | 0.499 | 1.115(0.581-2.141) | 0.743 |
| ECOG 3-4vs.0-2 | 0.969(0.562-1.670) | 0.910 | 0.875(0.458-1.673) | 0.687 |
| Bone marrow blast  > 60vs. ≤60 | 0.485(0.276-0.850) | **0.011** | 0.490(0.251-0.958) | **0.037** |
| ELN risk group  adverse vs non-adverse | 3.473(1.971-6.117) | **<0.001** | 2.731(1.386-5.382) | **0.004** |
| Achieved CR/CRi | 0.184(0.102-0.331) | **<0.001** |  |  |
| Methylation level  (continuous variable) | 0.972(0.954-0.992) | **0.005** | 0.962(0.939-0.987) | **0.003** |
| DNMT3A | 0.870(0.409-1.850) | 0.717 | 1.067(0.468-2.433) | 0.877 |
| IDH1 | 0.786(0.283-2.183) | 0.644 | 0.812(0.286-2.300) | 0.695 |
| IDH2 | 0.607(0.323-1.141) | 0.121 | 0.623(0.300-1.294) | 0.204 |
| JAK2 | 0.735(0.101-5.334) | 0.760 | 0.886(0.121-6.493) | 0.905 |
| TP53 | 3.409(1.776-6.544) | **<0.001** | 6.919(2.333-20.517) | **<0.001** |
| ASXL1 | 0.944(0.443-2.010) | 0.880 | 0.798(0.332-1.915) | 0.613 |
| FLT3-ITD | 0.431(0.171-1.085) | 0.074 | 0.716(0.298-1.719) | 0.455 |
| FLT3-TKD | 0.747(0.319-1.752) | 0.503 | 1.382(0.631-3.025) | 0.419 |
| NPM1 | 0.342(0.145-0.806) | 0.014 | 0.641(0.301-1.366) | 0.250 |
| STAG2 | 3.643(1.400-9.483) | 0.008 | 2.862(0.674-12.162) | 0.154 |
| TET2 | 1.008(0.503-2.021) | 0.981 | 1.401(0.651-3.013) | 0.389 |
| NF1 | 0.931(0.290-2.991) | 0.904 | 0.436(0.060-3.185) | 0.413 |
| SRSF2 | 1.626(0.726-3.641) | 0.237 | 1.921(0.670-5.506) | 0.224 |
| KIT | 0.478(0.066-3.467) | 0.465 | 1.707(0.523-5.572) | 0.376 |
| CEBPA | 0.580(0.180-1.867) | 0.361 | 1.187(0.462-3.050) | 0.723 |
| CEBPA-bZIP | 0.446(0.108-1.846) | 0.265 | 0.483(0.116-2.018) | 0.318 |
| PTPN11 | 1.280(0.310-5.282) | 0.733 | 1.420(0.340-5.930) | 0.630 |
| RUNX1 | 1.361(0.680-2.723) | 0.384 | 1.502(0.653-3.455) | 0.338 |
| K/NRAS | 1.186(0.577-2.435) | 0.643 | 1.466(0.690-3.111) | 0.320 |
| EZH2 | 0.295(0.041-2.140) | 0.227 | 0.330(0.045-2.412) | 0.275 |
| U2AF1 | 4.041(0.945-17.291) | 0.060 | 2.223(0.292-16.942) | 0.441 |
| BCOR | 0.764(0.238-2.456) | 0.651 | 0.728(0.175-3.028) | 0.662 |
