# Supplementary material for: miR-182 promoter hypermethylation predicts the better outcome of AML patients treated with AZA + VEN in a real-world setting
Source: Clin Epigenetics. 2025 Feb 5;17:18. doi: 10.1186/s13148-025-01823-1 (PMC11800541; doi:10.1186/s13148-025-01823-1)
Supplement: Supplementary file 9 — Additional file 9. [file 13148_2025_1823_MOESM9_ESM.docx]

Table S3. Multivariable analysis of clinical factors for survival in AML patients

|  | Overall survival  HR (95% CI) | p-value | Leukemia-free survival HR (95% CI) | p-value |
| --- | --- | --- | --- | --- |
| Bone marrow blast  ＞ 60 vs. ≤ 60 | 0.627(0.320-1.227) | 0.173 | 0.616(0.303-1.252) | 0.181 |
| ELN risk group  adverse vs. non-adverse | 2.472(1.257-4.864) | **0.009** | 2.444(1.212-4.930) | **0.013** |
| Achieved CR/CRi | 0.322(0.155-0.668) | **0.002** |  |  |
| Methylation level  (continuous variable) | 0.968(0.947-0.990) | **0.004** | 0.969(0.946-0.991) | **0.007** |
| TP53 | 0.990(0.418-2.342) | 0.981 | 2.979(0.966-9.191) | 0.058 |
| STAG2 | 2.407(0.728-7.959) | 0.150 |  |  |
| NPM1 | 0.614(0.228-1.656) | 0.336 |  |  |
